# α-Synuclein and Neuronal Cell Death

**DOI:** 10.1007/s12035-012-8327-0

**Published:** 2012-08-31

**Authors:** Toru Yasuda, Yasuto Nakata, Hideki Mochizuki

**Affiliations:** 1 Department of Neurology, Osaka University Graduate School of Medicine, 2-2 Yamadaoka, Suita, Osaka, 565-0871 Japan; 2 Department of Neurology, Kitasato University School of Medicine, Kanagawa, Japan; 3 Division of Neuroregenerative Medicine, Kitasato University School of Medicine, Kanagawa, Japan

**Keywords:** α-Synuclein, Apoptosis, Dopaminergic neuron, Neuroprotection, Parkinson’s disease, Substantia nigra

## Abstract

Parkinson’s disease (PD) is a progressive neurodegenerative disorder affecting ∼1 % of people over the age of 65. Neuropathological hallmarks of PD are prominent loss of dopaminergic (DA) neurons in the substantia nigra and formation of intraneuronal protein inclusions termed Lewy bodies, composed mainly of α-synuclein (αSyn). Missense mutations in *αSyn* gene giving rise to production of degradation-resistant mutant proteins or multiplication of wild-type *αSyn* gene allele can cause rare inherited forms of PD. Therefore, the existence of abnormally high amount of αSyn protein is considered responsible for the DA neuronal death in PD. Normally, αSyn protein localizes to presynaptic terminals of neuronal cells, regulating the neurotransmitter release through the modulation of assembly of soluble *N*-ethylmaleimide-sensitive factor attachment protein receptor complex. On the other hand, of note, pathological examinations on the recipient patients of fetal nigral transplants provided a prion-like cell-to-cell transmission hypothesis for abnormal αSyn. The extracellular αSyn fibrils can internalize to the cells and enhance intracellular formation of protein inclusions, thereby reducing cell viability. These findings suggest that effective removal of abnormal species of αSyn in the extracellular space as well as intracellular compartments can be of therapeutic relevance. In this review, we will focus on αSyn-triggered neuronal cell death and provide possible disease-modifying therapies targeting abnormally accumulating αSyn.

## Introduction

**Fig. 1:**
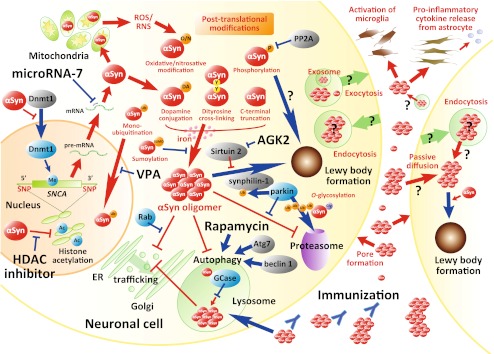
Schematic representation of molecular events and potential therapeutic targets associated with abnormal αSyn in PD. The molecular events that are reduced in PD and/or potentially neuroprotective, or considered to be neurotoxic, are shown in *blue*, or *red arrows* and *inhibitory lines*, respectively. Accumulation of αSyn oligomer, which can be modulated by several post-translational modification(s) of αSyn, leads to reduced neuronal cell viability by inhibiting ER-Golgi trafficking, autophagy, and/or proteasome. Mitochondrial translocation of αSyn induces production of ROS and RNS, further enhancing oxidative/nitrosative modification of αSyn. Oligomerized αSyn species can also be secreted into extracellular space, which might induce inflammatory glial reactions, pore formation on plasma membrane, or transmission to the neighboring neuronal cells to promote Lewy formation and/or cell death. These neurotoxic events can be ameliorated by several ways as indicated (also see the main text)

Parkinson’s disease (PD) is an age-related and the second most common neurodegenerative disorder beyond Alzheimer’s disease [1]. Clinical manifestation of PD is typical movement abnormalities that include resting tremor, rigidity, bradykinesia/akinesia, and postural instability. Neuropathological hallmarks in PD brains are (1) a prominent loss of dopaminergic (DA) neurons in the substantia nigra (SN) pars compacta (SNpc) projecting into the caudate/putamen (collectively called as striatum), and (2) formation of protein inclusions termed Lewy bodies and Lewy neurites that can be found in neuronal somas and processes, respectively. These aggregates are composed mainly of α-synuclein (αSyn) protein [2, 3]. Severe deprivation of striatal dopamine in PD can most effectively be treated with oral administration of dopamine precursor levodopa, whereas a long-term and pulsatile treatment with levodopa gradually induces adverse involuntary movements such as motor fluctuations and dyskinesias [4]. On the other hand, neurosurgical procedures including deep brain stimulation can partially normalize neuronal activities that have been agitated by the loss of the nigrostriatal DA pathway [5]. However, there have been no therapeutic options available that can reverse or even retard the progression of the disease, and such treatments are urgently required. To date, numerous efforts have been concentrated to elucidate the molecular mechanisms underlying the DA cell death in PD. In this article, we will review the relationship between abnormal αSyn and neuronal cell death. Several key molecules that can modulate the αSyn-induced neuronal death have hitherto been identified and investigated in αSyn-related animal models. We will also discuss such neuroprotective remedies for potential clinical interventions in PD (summarized in Fig. 1).

## Neuronal Cell Death in PD Brains: Apoptotic or Non-apoptotic?

The way in which DA neurons die is the principal enigma in the field of PD research. In neurodegenerative environments, neurons die through distinct fashions that are distinguished by morphological features: (1) apoptosis (known as type 1 cell death) [6–16], (2) autophagy (type 2 cell death) [9–11], and (3) necrosis (type 3 cell death) or “necroptosis” [12–16]. Apoptosis is evolutionally conserved cell-suicide mechanism indispensable for fundamental biological processes such as normal development, elimination of malignant neoplasms, and establishment of neuronal circuitry [6]. The morphologic features of apoptosis include nuclear and cytoplasmic condensation, internucleosomal DNA cleavage, and packaging of the dying cell into apoptotic bodies that are engulfed by phagocytes, preventing release of intracellular components [7]. Pathogenic apoptosis cascade can be induced by (1) mitochondrial damage that involves B cell lymphoma 2 (Bcl-2) family proteins, apoptotic protease-activating factor 1 (apaf-1), and the cysteine proteases caspases (referred to as intrinsic pathway); and (2) agonistic ligands of death receptors such as tumor necrosis factor α (TNFα), Fas ligand (FasL), and TNF-related apoptosis-inducing ligand (TRAIL), which promote activation of caspase-8 inside the cell (extrinsic pathway) [10]. The involvement of apoptotic cascade in DA neuronal death has been controversial in PD [17–25]. We and other groups have previously reported the positive staining of DA neurons in PD for terminal deoxynucleotidyl transferase dUTP nick end labeling (TUNEL) and chromatin condensation, which is the typical process seen in apoptotic cell death [17, 21, 22]. However, other groups found no signs of apoptosis in the nigral DA neurons, regardless of disease duration, severity, drug treatment, and age of the patient [19, 20]. Using electron microscopy, Anglade et al. [18] showed the presence of condensed chromatin in the nucleus of neuromelanin-containing neurons and engulfment of apoptotic bodies in glial cells. Importantly, they also observed cells displaying the features of autophagic degeneration, implying that apoptosis may not be the sole form involved in DA neuronal death [18].

Autophagy is an evolutionally conserved mechanism for a bulk degradation of cellular components, including proteins and organelles, and serves as a cell survival mechanism during nutrient deprivation [9]. There exists a complex crosstalk between apoptosis and autophagy [10]. Common upstream signals sometimes result in combined autophagy and apoptosis at the single cell level. In other instances, the cell dictates autophagy or apoptosis in a mutually exclusive manner. Under certain circumstances, autophagy allows cells to adapt to stress, thereby avoiding apoptotic cell death, e.g., a harmful αSyn can be degraded by autophagic pathway (see below). By contrast, massive autophagy induces alternative cell death pathway that is called autophagic cell death (ACD) [9, 10]. ACD is characterized by the presence of autophagic vacuoles (autophagosomes), which can be identified as double-membraned vesicles, and autophagolysosomes, which arise from the fusion of autophagosomes and lysosomes and are defined by a single membrane, in dying cells [9, 10]. On the other hand, Kroemer and Levine [11] indicated that the term ACD may be a misnomer because that is, in many cases, cell death “with” autophagy rather than cell death “by” autophagy. They emphasized that the autophagic process is not the executioner of cell death, or rather, cytoprotective response under pro-apoptotic condition [11].

Energy depletion is a potent trigger of necrosis [13]. Morphologically, necrosis is characterized by extensive vacuolation of the cytoplasm, mitochondrial swelling, dilatation of the endoplasmic reticulum (ER) and nuclear membrane, condensation of chromatin into small, irregular, and circumscribed patches, and plasma membrane rupture. Necrotic cells are lysed and do not fragment into discrete corpses as their apoptotic counterparts do. As a consequence, cellular contents are liberated into the extracellular space, which might precipitate damage to neighboring cells and evoke inflammatory responses [13, 15]. Necrosis has traditionally been considered merely as an accidental, uncontrolled form of cell death that only occurs in pathological conditions. Also, apoptosis has long been believed the sole form of programmed cell death (PCD). However, accumulating evidence uncovered another route of PCD, a programmed necrosis termed necroptosis [reviewed in 14, 15]. While several articles have suggested the occurrence of the “non-apoptotic PCD” during neurodegenerative processes [12, 14, 15, 26, 27], there have been a limited number of reports documenting the necrotic cell death in PD brains. This might in part be attributed to a methodological difficulty to dissect necrotic cell explosion in the postmortem brain tissues. It is known that necroptosis is triggered by ligation of death receptors with TNFα, FasL, and TRAIL, the same ligands that activate apoptosis [14, 15]. A death domain-containing kinase receptor-interacting protein 1 (RIP1) and RIP3 are required to dictate necroptotic pathway. Caspase-8 inactivates RIP1 and RIP3 by proteolytic cleavage and initiates the pro-apoptotic caspase activation cascade [15]. By contrast, inhibition of caspase-8 results in execution of the programmed necrosis in primary DA cultures [16]. A small molecule inhibitor of necroptosis, necrostatin-1, attenuated RIP1 kinase activity [28] and prevented glutamate-induced hippocampal neuronal cell death [29]. It needs further explorations to determine the involvement of necroptosis in DA neuronal degeneration in PD.

## Physiological Functions of αSyn

αSyn is a neuronal protein of 140 amino acids and normally localized to presynaptic terminals. The exact physiological function of αSyn remains yet defined, but several works have implicated its role in dopamine biosynthesis, synaptic plasticity, and vesicle dynamics [1, 30–32]. Indeed, αSyn directly binds to vesicle-associated membrane protein 2 (VAMP2; also called as synaptobrevin-2) and promotes assembly of soluble *N*-ethylmaleimide-sensitive factor attachment protein receptor (SNARE) complex through a nonclassical chaperone activity [33]. Orchestration of assembly/disassembly of SNARE complex is essential for the regulation of neurotransmission. Recent studies have implicated presynaptic dysfunction to be an initial event of neurodegeneration [34]. A presynaptic protein cysteine-string protein-α (CSPα) also promotes SNARE complex assembly through the formation of chaperone complex with heat shock cognate 70 (Hsc70) and the small glutamine-rich protein SGT [35, 36]. The CSPα-Hsc70-SGT complex binds directly to synaptosomal-associated protein of 25 kDa (SNAP-25), whereby promoting SNARE complex formation [36]. Depletion of CSPα in mice represents decreased level of SNAP-25 and corresponding reduced assembly of SNARE complex [36]. Intriguingly, the CSPα-knockout mice show a rapidly progressive neurodegeneration and premature death, both of phenotype counteracted by transgenic expression of αSyn [37]. On the other hand, increased expression of αSyn in the absence of overt cell toxicity markedly inhibited neurotransmitter release, which was attributed to a perturbed synaptic vesicle density at the active zone, due to a defective reclustering of synaptic vesicles after endocytosis [38]. In another study, overexpressed αSyn indirectly inhibited SNARE-mediated exocytosis by sequestering arachidonic acid, which upregulates syntaxin and enhances its engagement with SNARE complex [39]. The opposing actions of αSyn implicate that a tight regulation of subcellular level and distribution of αSyn is indispensable for the intrinsic functions of neuronal cells.

## Pathogenic Roles of αSyn in PD

αSyn is one of the most extensively studied proteins in PD research [30–32, 40] (Fig. 1). The gene encoding αSyn (*SNCA*) is mutated in rare inherited forms of PD, resulting in amino acid substitutions (A53T [41], A30P [42], or E46K [43]; classified as PARK1), or multiplication of its allele (PARK4) [44, 45]. Moreover, αSyn is a major component of Lewy bodies and Lewy neurites found in sporadic cases [2, 3]. Therefore, the presence of abnormally high levels of αSyn protein due to unbalanced production and/or degradation is thought to trigger DA neuronal death in both familial and sporadic cases of PD (Fig. 1). Single nucleotide polymorphisms in the 5′-promoter and 3′-flanking regions of *SNCA* gene that influence αSyn protein level are associated with susceptibility to idiopathic PD [46–48]. Furthermore, genome-wide association studies identified *SNCA* as a common risk factor for PD [49, 50]. Recent two studies uncovered epigenetic regulation of *SNCA* gene expression. Reduced methylation in CpG islands at intron 1 of *SNCA* that leads to increased protein production was evident in the SN of sporadic patients with PD [51, 52]. Desplats et al. [53] showed reduction of nuclear level of DNA methyltransferase 1 (Dnmt1) and DNA methylation in human postmortem brains affected with PD and dementia with Lewy bodies (DLB). Physical association of αSyn with Dnmt1 might mediate the retention of Dnmt1 in the cytoplasm, which results in hypomethylation of DNA [53]. However, overexpressed αSyn protein sometimes functions as a neuroprotective molecule in cell types other than DA neurons [37, 54–56]. Also, a recent report indicated protective function of physiological level of αSyn in DA cells. In that study, αSyn was found to reduce p300/CBP level and its histone acetyltransferase activity, whereby suppressing the NFκB-mediated transcriptional expression of pro-apoptotic protein kinase Cδ [57]. Oxidative modification of αSyn by dopamine metabolites is considered responsible for the selective vulnerability to DA neurons [55, 58]. Dopamine-modified αSyn tends to form protofibrillar intermediates but not large fibrils [58]. Such “oligomeric” αSyn is supposed the real criminal in DA neuronal toxicity [59–66]. On the other hand, a recent important finding indicated that endogenous normal αSyn forms a helically folded tetrameric structure of 58 kDa in neuronal and non-neuronal cell lines, brain tissue, and human red blood cells [67]. The tetrameric αSyn had high lipid-binding capacity and little or no propensity for amyloid-like aggregation. They proposed that destabilization of the tetramer precedes the misfolding and aggregation of αSyn in pathogenic conditions with PD and other α-synucleinopathies [67]. Another group also indicated that bacterially produced αSyn forms a stable tetramer [68] (To avoid misconceptions, hereafter, the nomenclature “oligomer” will be applied for the toxic species of αSyn formed in the diseased situations).

A 22-kDa *O*-glycosylated form of αSyn (αSp22) is destined for proteasomal degradation by receiving polyubiquitin moieties through the action of E3 ligase parkin, which is linked to a recessively inherited young-onset PD, PARK2 [69, 70]. Overexpression of wild-type or familial PD-linked mutant of αSyn in cell culture impairs proteasome activity and induces apoptosis or ACD, depending on the experimental conditions [71–75]. Soluble oligomeric αSyn impaired proteasome activity and likely impeded access of other proteasomal substrates [76, 77]. αSyn is degraded not only via ubiquitin-proteasome system but also autophagy [78, 79]. Both macroautophagy and chaperone-mediated autophagy (CMA) are involved in the clearance of accumulating αSyn [80]. Overexpressed wild-type αSyn compromised macroautophagy by inhibiting Rab1a [81], and pathogenic mutant and dopamine-modified αSyn prevented their own degradation and that of other substrates in CMA [82, 83]. As a result, DA cells harboring abnormal αSyn are sensitized to degenerative stimuli.

The majority of cellular source of energy is produced in mitochondria in the form of ATP. Because of the electrons being transported along the respiratory chain to potentiate mitochondrial intermembranous proton gradient, the prerequisite for oxidative phosphorylation, this organelle can intrinsically be a primary source of reactive oxygen species (ROS). A number of studies have demonstrated mitochondrial dysfunction and oxidative (and nitrosative) stresses linked to neuronal cell degeneration in PD [reviewed in 84]. This is well illustrated in an animal model of PD generated with 1-methyl-4-phenyl-1,2,3,6-tetrahydropyridine (MPTP), which inhibits complex I in the electron transport system [85, 86]. αSyn protein has a non-canonical mitochondrial targeting sequence at its N-terminus and is indeed translocated to mitochondria in human fetal DA neuronal culture and postmortem normal brain tissues [87]. The mitochondrial αSyn accumulation is enhanced in PD brains. αSyn interacts with complex I and interferes with its function, promoting the production of ROS [87]. Particularly, superoxide radical rapidly reacts with nitric oxide to yield highly reactive peroxynitrite anion and ensuing reactive nitrogen species (RNS) [84]. ROS/RNS covalently modify lipids, nucleic acids, and proteins. αSyn can be modified with these compounds, augmenting the formation of toxic oligomeric αSyn (see below) [88].

Previous studies have implicated an increased iron level in the SN of postmortem brains of idiopathic PD and parkin-deficient PARK2 patients [89–91]. In MPTP-treated hemiparkinsonian monkeys, we and another group reported that DA cell death preceded iron accumulation [92, 93], suggesting that the elevation of iron may be a secondary event in nigral degeneration. On the contrary, several recent studies indicate that intraneuronal iron overload can be a primary cause of DA cell death in part through enhancing the formation of toxic radicals by Fenton reaction [94–96]. An iron transporter, divalent metal transporter 1 (DMT1), is upregulated and contributes to nigral DA neuronal death in MPTP and 6-hydroxydopamine rodent models of PD [94, 95]. Importantly, parkin regulates uptake of iron via degradation of DMT1 in ubiquitin-proteasome system [96]. These results suggest that DMT1-mediated iron overload can cause DA cell loss in parkinsonian brains. Iron promotes aggregation of αSyn protein [97, 98], and formation of pore-forming toxic oligomer species [99]. Moreover, DMT1-mediated cell death was aggravated in the presence of mutant αSyn as a result of excessive autophagic activity [100].

Recent studies revealed the association of Gaucher disease, the lysosomal storage disorder, with αSyn pathology [101–105]. Gaucher disease is caused by mutations in the gene encoding lysosomal protein glucocerebrosidase (GCase) that also increase the risk for PD and DLB [reviewed in 105]. A direct physical interaction between GCase and αSyn that prefers lysosomal acidic condition has been demonstrated [102]. In another study, importantly, glucosylceramide, which is the substrate of GCase and accumulated in Gaucher disease brains, directly influenced amyloid formation of αSyn by stabilizing soluble oligomeric intermediates [103]. The oligomeric αSyn in turn inhibited intracellular trafficking of GCase and decreased lysosomal GCase function. Such bidirectional effects of αSyn and GCase form a positive feedback loop that may lead to a self-propagating disease [103]. Genetic mouse model of Gaucher disease exhibited αSyn accumulation in the SN, cortex, or hippocampus [103, 104], and adeno-associated viral (AAV) vector-mediated delivery of GCase ameliorated pathological and behavioral aberrations in the Gaucher mice [104].

## Prion-Like Cell-to-Cell Transmission of αSyn

In the last 20 years, more than 300 patients with PD have received striatal transplantation of midbrain tissues that were isolated from aborted fetuses [106, 107]. Many of these patients experienced a transient improvement of motor symptoms [106, 107], while severe off-phase dyskinesia remains a major concern [108–110]. On the other hand, intriguingly, more than a decade after the fetal transplantation, Lewy-like inclusions were depicted to be present in the surviving DA cells in the grafts [111, 112]. These findings led to the current provocative hypothesis that αSyn protein itself might transmit from neuron to neuron like as prion proteins, whereby spreading the pathologies in the brains of PD and other α-synucleinopathies [113–115].

Indeed, αSyn and its oligomeric forms are localized in the lumen of vesicles in differentiated neuronal cells and rat synaptosomal preparations, and secreted via non-classical ER/Golgi-independent exocytosis like as a part of the normal life cycle of this protein [116]. The intravesicular αSyn was found more prone to aggregation compared with cytosolic αSyn [116]. Another group showed that soluble monomeric and oligomeric αSyn were externalized via the vesicles that have characteristic hallmarks of exosomes in a calcium-dependent manner, and significantly reduced cell viability [117]. Danzer et al. [63] showed that different species of extracellular αSyn oligomers can exert distinct effects on cells; some oligomeric αSyn induced cell death by presumably pore-forming mechanism, and the other form of oligomer directly entered the cell and enhanced aggregation of αSyn. They proposed that heterogeneous populations of oligomeric forms coexist in equilibrium [63]. A solution structure of the pore-forming αSyn oligomer has been determined by small angle X-ray scattering [65]. On the other hand, cationic liposome-mediated forced transduction of exogenously produced fibrils of αSyn could seed the intracellular formation of Lewy-like inclusion in cultured cells [118, 119]. Furthermore, several groups reported that the extracellular αSyn can be uptaken by cells through endocytotic mechanism, and the internalized αSyn enhanced aggregation of (endogenous or overexpressed) αSyn and neuronal cell death [120–122]. Importantly, Mougenot et al. [123] demonstrated prion-like propagation of αSyn pathology in αSyn-transgenic mice. Brain homogenates from old αSyn-transgenic mice, which display motor clinical signs and contain insoluble Ser129-phosphorylated αSyn, were intracerebrally inoculated to young αSyn-transgenic mice. This triggered an early onset of characteristic motor signs and a prominent formation of inclusions that contain Ser129-phosphorylated αSyn, compared with uninoculated αSyn-transgenic mice or mice inoculated with brain homogenate from young healthy αSyn-transgenic mouse [123]. In that experiment, αSyn-null mice showed no abnormalities when inoculated with the brain homogenate of old disease-state αSyn-transgenic mice, indicating the crucial role for the presence of (pre-abnormal) αSyn in the host brain [123]. Extracellular αSyn is also capable of inducing microglial activation [124] and pro-inflammatory cytokine release from astrocytes [125] that may enhance neuronal toxicity. Accordingly, removal of extracellular αSyn species may be relevant to disease modification. Vaccination or passive immunization targeting the overloaded αSyn has successfully cured mice from neuronal degeneration (see below) [126, 127].

## αSyn-Transgenic Animals

αSyn-transgenic models have been generated in mice [reviewed in 31, 128–130] and other organisms including nematode *Caenorhabditis elegans* [131–133] and fruit fly *Drosophila melanogaster* [134, 135]. Nematode models of αSyn overexpression exhibited neuronal or dendritic loss of DA cells and corresponding behavioral deficits [131–133]. *Drosophila* models of αSyn overexpression show adult-onset loss of DA neurons and locomotor dysfunction [134, 135]. These invertebrate models well recapitulate several key features of human PD and are relevant for comprehensive genetic analyses and drug screening towards elucidating the molecular pathogenesis and developing therapies for α-synucleinopathies [131–135].

On the other hand, a single transgenic expression of wild-type or familial PD-associated αSyn mutant in mice hardly represents a progressive loss of DA cells in the SNpc [31, 128–130]. Masliah et al. [136] reported the decrease of the striatal DA terminals and corresponding motor impairment induced by the overexpression of wild-type αSyn under the regulatory control of human platelet-derived growth factor-β (PDGF-β) promoter. Thereafter, several lines of αSyn-transgenic mice were generated and displayed severe movement disorders, loss of neuronal cells other than DA ones, and/or synaptic dysfunction before overt neuronal loss. In the transgenic mice of αSyn A53T mutant driven by the mouse prion promoter, which were originally reported by Lee et al. [137], Martin et al. [138] found that neocortical, brainstem, and motor neurons developed Lewy-like intraneuronal inclusions, axonal degeneration, and mitochondrial damage, as well as p53- and caspase-3-mediated apoptotic death. This report provided a mechanistic insight into the severe movement disorder of the αSyn A53T-transgenic mice. Sotiriou et al. [140] recently showed that the mouse prion promoter-αSyn A53T-transgenic mice, originally reported by Giasson et al. [139], had selective vulnerability for noradrenergic systems in the spinal cord, olfactory bulb (OB), and striatum in an age-dependent manner, while DA cells in the SN and noradrenergic cells in the locus coeruleus were not affected [140]. Lim et al. [141] generated inducible line of αSyn-transgenic mice with a tet-off system and the calcium/calmodulin-dependent protein kinase IIα (CaMKIIα) promoter, in which A53T mutant can be conditionally expressed in neuronal cells mainly in the cortex and hippocampus, to model human DLB. αSyn pathology and age-dependent neuronal cell loss was observed in cortical and hippocampal areas that resulted in memory impairment. Drug-induced suppression of αSyn transgene partially cleared pre-existing αSyn pathology and reverted defects in presynaptic proteins including synaptophysin, CSPα, synaptotagmin, SNAP-25, and syntaxin, and corresponding memory functions [141]. These results emphasize that targeted removal of αSyn pathology can reverse cognitive decline in DLB.

On the other hand, oxidation and nitration of αSyn induces the formation of stable dimers and oligomers through intermolecular dityrosine cross-linking [88]. αSyn possesses four tyrosine residues at positions 39, 125, 133, and 136 and lacks cysteine. When cysteine was substituted for tyrosine 39 and 125, these mutants increased intracellular inclusions and induced apoptosis in a rat DA cell line [142]. They indicated that cross-linking at critical positions in αSyn molecule can increase dimer formation, and accelerate protein aggregation and cellular toxicity of αSyn [142]. αSyn-transgenic mice carrying Y39C substitution under the murine Thy1 promoter were then generated and analyzed [143]. The mice showed age-dependent formation of αSyn oligomer and aggregate, progressive apoptotic cell loss in the cortex, and motor and cognitive deficits similar to DLB. Midbrain DA neurons and spontaneous locomotor activity were not affected in the αSyn Y39C-transgenic mice [143].

The murine prion promoter-αSyn-transgenic mice carrying E46K mutation, initially reported to cause PD and DLB [43], displayed detrimental age-dependent motor impairment, although DA neurons in the SN did not produce αSyn E46K protein [144]. These animals accumulated intracytoplasmic neuronal inclusions of αSyn in the cerebellum and pons that more closely resemble nigral Lewy bodies in PD than the previously described transgenic mice of human A53T αSyn. Intriguingly, phosphorylated tau-positive inclusions were found in the motor cortex and pons of the αSyn E46K-transgenic mice [144].

αSyn can be processed by C-terminal truncation in normal and PD brains [145, 146], and this modification promotes aggregation of αSyn [145–149]. The transgenic mice, that express C-terminally truncated form of αSyn [αSyn(1–120)] under the control of rat tyrosine hydroxylase (TH) promoter on a mouse αSyn-null background, exhibited the formation of pathological αSyn-positive inclusions in the SN and OB, reduction of the striatal dopamine levels, and a progressive reduction in spontaneous locomotion, in the absence of DA cell death [150]. In the following study, they investigated the presynaptic SNARE proteins in the striatum of the αSyn(1–120)-transgenic mice [151]. Synaptic accumulation of αSyn was accompanied by age-dependent redistribution of SNAP-25, syntaxin-1, and synaptobrevin-2, as well as reduced exocytosis of dopamine. A similar redistribution of the SNARE proteins was detected in PD brains [151]. Of note, Wakamatsu et al. [152] reported a selective loss of DA neurons in the SNpc of the transgenic mice carrying human αSyn(1–130). This truncated form of αSyn further caused reduction of the striatal DA axon terminals and dopamine level with corresponding reduction of locomotor activity, which can be reversed by administration of levodopa. However, the loss of nigral DA neurons was not progressive and seemed to occur during embryogenesis along with the onset of transgene expression [152].

Mutations in *leucine-rich repeat kinase 2* (*LRRK2*) gene have been linked not only to a dominantly inherited PARK8 [153, 154] but also to sporadic form of PD [155]. The gene product LRRK2 possesses multiple functional domains including GTPase and kinase domains [156, 157]. A commonly found mutation, G2019S, increased its kinase activity, suggesting a gain-of-function mechanism for the pathogenesis of LRRK2-linked PD [157]. Intriguingly, LRRK2 accelerated the progression of neuropathology of αSyn [158]. Lin et al. [158] produced inducible line of LRRK2- or αSyn A53T-transgenic mice with a tet-off system and CaMKIIα promoter, in which the PD-related transgene can be expressed at high-level (LRRK2: about 8- to 16-fold; and αSyn A53T: about 30-fold) in neuronal cells in the striatum and cortex. While LRRK2 alone did not cause neurodegeneration, the presence of excess LRRK2 G2019S exacerbated abnormal accumulation and aggregation of αSyn A53T, which likely stemmed from the impairment of microtubule dynamics, Golgi organization, and the ubiquitin-proteasome pathway. Morphological abnormality of mitochondria and superoxide production was also promoted in the presence of high amount of LRRK2. In their αSyn A53T mice, genetic ablation of LRRK2 preserved the Golgi structure and suppressed the accumulation/aggregation of αSyn, and then delayed the progression of neuropathology [158]. This study elegantly demonstrated that suppression of LRRK2 can be a potential therapeutic target to ameliorate αSyn-induced neurodegeneration. In another report, by contrast, a single LRRK2-knockout mouse, which has a normal nigrostriatal DA system, developed accumulation and aggregation of αSyn and ubiquitinated proteins in the kidneys during aging [159]. This was possibly due to impairment of autophagy-lysosomal pathway. Furthermore, the ablation of *LRRK2* gene dramatically increased apoptotic cell death, inflammatory responses, and oxidative damage in the kidney. These mice implicated that loss-of-function mutations of LRRK2 may cause cell death via impairment of protein degradation pathways, which lead to αSyn accumulation and aggregation [159].

## Viral Vector-Mediated αSyn Overexpression Models

**Fig. 2:**
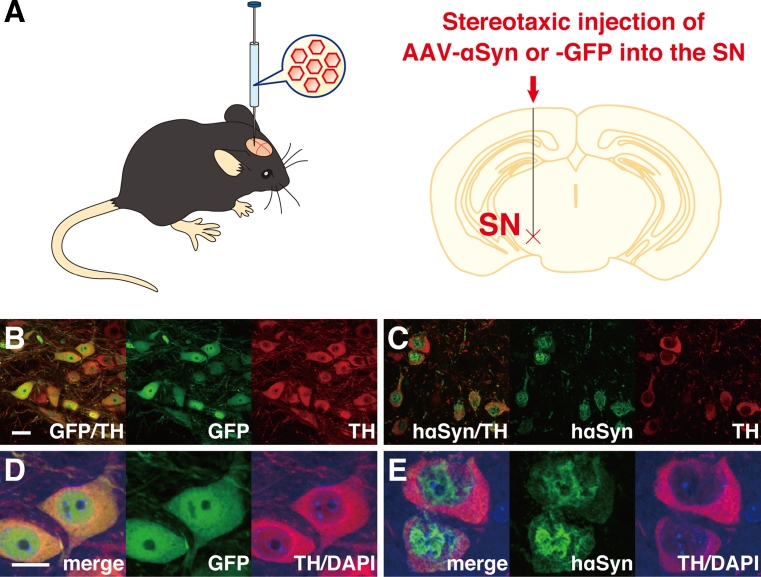
AAV vector-mediated expression of foreign gene in mouse brain. **a** The AAV vector can be injected stereotaxically into the SN of mice. **b**–**e** Representative images for the AAV vector-mediated overexpression of human αSyn (hαSyn) or GFP in DA cells. Nigral sections of the AAV-GFP- (**b**, **d**) or AAV-hαSyn-injected mice (**c**, **e**) were immunostained for GFP (GFP; **b**, **d**; shown in *green*) or hαSyn (**c**, **e**; *green*) and tyrosine hydroxylase (TH; **b**–**e**; *red*; merged with anti-GFP or hαSyn, *yellow*). Images for DAPI are also merged (**d**, **e**; *blue*). Note that the overexpression of hαSyn caused a profound loss of DA cell bodies with neuritic pathology. The overexpressed hαSyn was localized to nucleus and cytoplasm in a heterogeneous pattern in the remaining DA cells, while GFP distributed uniformly. *Scale bars*: (**b**, **c**) 20 μm and (**d**, **e**) 10 μm

PD models have also been generated by viral vector-mediated overexpression of αSyn in rodents and nonhuman primates [160–166]. The AAV and lentiviral vectors have efficient tropism for DA neurons when injected into the SN and the ability of long-term stable gene expression with low accompanying cytotoxicity [167, 168]. In rodents, the viral vector-mediated overexpression of wild-type and familial PD-associated αSyn mutants can cause a progressive loss of DA cell bodies with neuritic pathology [160–163]. Representative images are shown in Fig. 2. The DA cell death was accompanied with phosphorylation of αSyn at Ser129 residue and apoptotic cascade with activation of caspase-9 in our examination [163]. We have also reported that, in the presence of the PARK5-linked ubiquitin carboxy-terminal hydrolase-L1 (UCH-L1) I93M mutant in mice, the AAV-αSyn-induced accumulation of αSyn and apoptotic DA cell death was enhanced, but not influenced in the absence of wild-type UCH-L1, indicating that PARK5-linked PD might be caused by gain-of-function mutation in UCH-L1 [166]. Importantly, Chung et al. [169] found that disturbance of the proteins relevant to synaptic transmission and axonal transport preceded the AAV-αSyn A53T-induced DA neuronal loss. It is known that a majority of the virally αSyn-challenged rodents lacks significant behavioral abnormalities, although they finally exhibit a profound DA neurodegeneration [160, 161, 163].

By contrast, adult common marmosets (*Callithrix jacchus*) injected with αSyn-encoding AAV exhibited a severe neuronal pathology with a significant motor impairment such as head position bias in a short-term (16-weeks) study [164]. In a long-term examination for 1 year, the αSyn-treated monkey displayed behavioral impairments including full body rotation, head turn bias, and slowed and decreased use of contralateral hand [165]. These motor abnormalities were most pronounced in αSyn A53T-transduced group compared with wild-type αSyn and control GFP groups. About half of the αSyn A53T monkeys analyzed further developed slips of contralateral limbs (hand and foot) and persistent head tilts down on the contralesional side in the later phase [165]. Pathologically, wild-type αSyn-transduced monkeys exhibited a notably lower density of fibers immunopositive for αSyn in the caudate and putamen than for GFP in the GFP-transduced monkeys. The αSyn-containing aggregates were also found in the striatal fibers. This finding was even more pronounced in the αSyn A53T group, where only a sparse network of αSyn-positive fibers was seen in the caudate/putamen. In the αSyn A53T group, the ectopic αSyn protein appeared to have cleared from the SN, and there were fewer surviving αSyn-positive cell bodies compared with wild-type αSyn and GFP groups. When the Ser129-phosphorylated αSyn was examined by immunostaining, some of the neurons in the SN appeared normal while other cells were atrophic with shrunken cell bodies or had dystrophic dendrites, some with beaded aggregations. Interestingly, in several cases, the Ser129-phosphorylated αSyn-positive staining was localized to the nucleus (see below) [165]. In the wild-type and A53T αSyn groups, a substantial loss of TH-positive DA axon terminals and numerous pathological TH-positive accumulations were found in the striatum, suggesting that some of the affected but surviving cells were nonetheless dysfunctional. In the SN, the αSyn A53T-transduced monkeys showed a clear and consistent DA neurodegeneration in the injected side, which was significantly different when compared with GFP and wild-type αSyn groups [165]. The nonhuman primate model of α-synucleinopathy will be greatly useful for preclinical researches potentially preventing or retarding the behavioral and pathological progressions of the disease.

## Phosphorylation and Neurotoxicity of αSyn

As described above, αSyn receives several post-translational modifications in diseased brains. In particular, Fujiwara et al. [170] found that about 90 % of αSyn proteins deposited in the brains of α-synucleinopathy are phosphorylated at Ser129 residue. Thereafter, the relationship between phosphorylation and neuronal toxicity of αSyn has been investigated. In dopamine-producing cells, rotenone treatment induced Ser129 phosphorylation of αSyn and formation of Lewy-like aggregates, with increased apoptotic cell death through the unfolded protein response [171]. In another study, increased oxidative stress or proteasomal inhibition caused significant elevation of soluble and non-aggregated form of Ser129-phosphorylated αSyn with increased DA cell death [172]. These in vitro studies suggested that Ser129 phosphorylation of αSyn is toxic to DA cells. Chen and Feany [173] reported that phosphorylation at Ser129 is essential for αSyn to have neuronal toxicity in a *Drosophila* model of PD. The toxicity was abolished by amino acid substitution S129A that is no longer phosphorylated, and reproduced by S129D that carries a negative charge mimicking phosphate on serine residue [173]. On the other hand, phosphoprotein phosphatase 2A (PP2A) dephosphorylates αSyn at Ser129, and this activity is enhanced by carboxyl methylation of the catalytic C subunit of PP2A [174]. αSyn-transgenic mice raised on a diet supplemented with eicosanoyl-5-hydroxytryptamide, an agent that enhances PP2A methylation, dramatically reduced both Ser129 phosphorylation and aggregation of αSyn in the brain [174]. These mice displayed enhanced neuronal activity, increased dendritic arborizations, and reduced astroglial and microglial activation, as well as improved motor performance [174].

There exist opposing reports as to the neurotoxicity of the Ser129-phosphorylated αSyn in the viral vector-mediated rodent model of αSyn overexpression. Alteration of Ser129 to nonphosphorylated Ala resulted in enhanced [175, 176] or unchanged toxicity of αSyn [177], and alteration of Ser129 to a phospho-mimetic Asp resulted in eliminated [175, 176] or unchanged toxicity of αSyn [177]. These studies suggest that the Ser129 phosphorylation of αSyn has, if any, protective effect on DA neurons. We recently reported that viral vector-mediated delivery of parkin prevented DA neuronal loss induced by a chronic MPTP in mice [178]. The osmotic minipump-mediated MPTP infusion caused accumulation of the Ser129-phosphorylated αSyn in DA cells, which was enhanced by overexpression of parkin, suggesting that the phosphorylation resulted in reduced toxicity of αSyn [178]. This result is in line with the report by Lo Bianco et al. [179] who demonstrated that lentiviral-parkin attenuated αSyn-induced DA cell loss by increasing the number of the Ser129-phosphorylated αSyn-positive inclusions in rats. The discrepancy in the neurotoxic consequence of the αSyn Ser129 phosphorylation makes difficulties in developing disease-modifying therapies. More elaborate time-series examinations in primates might be required to target this post-translational modification.

## Prevention of αSyn-Induced Neuronal Cell Death/Dysfunction

αSyn-induced neuronal cell death and dysfunction can be targeted by several strategies. Masliah’s group has reported effective treatment of αSyn-transgenic mice with active and passive immunization protocols, which enabled clearance of toxic αSyn in multiple neuronal populations simultaneously [126, 127]. Passive immunization with a monoclonal antibody directed against C-terminus of αSyn (epitope: 118–126 amino acids of αSyn) that crossed into the central nervous system ameliorated behavioral deficits and synaptic abnormalities in αSyn-transgenic mice [127]. Moreover, the monoclonal antibody reduced the accumulation of calpain-cleaved and oligomerized αSyn aggregates in neuronal cells via lysosomal-degradation pathway [127]. They further indicated that lentiviral vector-mediated transduction of beclin 1, a regulator of autophagic pathway, ameliorated the synaptic and dendritic pathology in αSyn-transgenic mice [79]. The reduced accumulation of αSyn induced by the beclin 1 transduction was accompanied by enhanced lysosomal activation. These studies demonstrated that beclin 1-mediated autophagy pathway plays an important role in the intracellular degradation of αSyn and may present a novel therapeutic target for DLB and PD [79].

A number of studies demonstrated that parkin, PARK2-associated ubiquitin E3 ligase, protects against αSyn-induced cell death in vitro. Petrucelli et al. [72] showed that αSyn A53T-mediated toxicity in primary neuronal culture, which could be mimicked by the application of proteasome inhibitor, was reduced by E3 ligase activity of parkin. This study implicated that parkin and αSyn are linked in a common pathway associated with selective DA neuronal cell death. Another group reported that parkin could restore the reduced cell viability induced by wild-type αSyn via activation of calpain [180]. The calpain-mediated cleavage of accumulated αSyn occurred independently of proteasomal degradation [180]. In *Drosophila* model of PD, parkin suppressed DA neuronal death induced by overproduction of αSyn as well as parkin-associated endothelin receptor-like receptor (Pael-R) [181]. In rats, we have shown that AAV vector-mediated parkin delivery ameliorated DA cell loss induced by overexpression of wild-type αSyn [182]. On the other hand, parkin is known to interact with and ubiquitinate synphilin-1 [183], which was isolated as αSyn-interacting protein by yeast two-hybrid screen [184], through nonclassical K63-linked fashion [185]. Co-expression of αSyn and synphilin-1 resulted in the formation of Lewy body-like ubiquitin-positive cytosolic inclusions [183–185], which were found to be cytoprotective under proapoptotic stimuli [186]. A recent study indicated that transgenic expression of synphilin-1 attenuated αSyn-induced cell death in mice [187]. A double-transgenic mouse for αSyn A53T-mutant and synphilin-1 exhibited longer lifespan, improved motor performance, and reduced neuronal degeneration in the brainstem as compared to their single αSyn A53T-transgenic counterparts. Increased expression of beclin 1 and enhanced formation of aggresome-like structures were observed in the double αSyn A53T/synphilin-1-transgenic mice [187]. On the other hand, αSyn can directly be modified with small ubiquitin-related modifier (SUMO) at the positions of lysine 96 and 102 residues [188, 189]. The sumoylated αSyn showed increased solubility, whereas unmodified αSyn formed fibrils. Simultaneous substitution of K96 and K102 to arginine residues, which significantly impaired the sumoylation but did not affect the ubiquitination status of αSyn, was manifested by increased aggregation propensity and neuronal toxicity in vitro and in vivo [189]. Regulation of αSyn sumoylation may thus have a therapeutic potential.

We have shown previously that downregulation of *SNCA* transcripts by the AAV-mediated transduction of ribozymes provided rat DA neurons with a resistance to neurotoxin-induced αSyn accumulation and cell death [190]. Recently, Junn et al. [191] reported that downregulation of αSyn expression via microRNA-7 was effective for protection of αSyn A53T-expressing cells against oxidative stress. MicroRNA-7 was abundantly expressed in neurons in the SN, striatum, and OB in mice, the most affected areas in PD. Intoxication of mice with MPTP caused 50 % decrease of microRNA-7 in ventral midbrain, raising the possibility that the reduction of microRNA-7 in PD may cause degeneration of the nigrostriatal system, likely through upregulating αSyn production [191].

Sirtuin family of the class III NAD^+^-dependent histone deacetylases (HDACs) is involved in a variety of biological processes and several age-associated diseases [192, 193]. One of the family members, sirtuin 1, the mammalian ortholog of yeast Sir2, is upregulated under the conditions of caloric restriction and resveratrol treatment, and has a critical role in cell survival [192, 193]. On the other hand, sirtuin 2 induces neuronal cell death through its protein deacetylase activity [194]. The opposing mode of function is called as yin and yang of sirtuins [195]. A potent inhibitor of the deacetylase activity of sirtuin 2, AGK2, alleviated αSyn-induced DA neuronal cell death in primary cell culture and *Drosophila* models of PD [194]. In αSyn-aggregation experiment, where αSyn and synphilin-1 were co-introduced, AGK2 decreased the number and increased the size of αSyn aggregates, suggesting that the formation of large aggregates of αSyn might affect neuronal survival [194]. On the other hand, αSyn was found localized to the nucleus of DA neurons in mice that were exposed to neurotoxic herbicide paraquat, and associated with histones in vitro [196]. Kontopoulos et al. [197] have shown in DA cell line and *Drosophila* models that wild-type αSyn, C-terminally tagged with nuclear localization sequence or nuclear export sequence, enhanced or attenuated the neuronal toxicity, respectively. The inherited PD-linked A53T or A30P mutation promoted the nuclear localization of αSyn. Intranuclear αSyn inhibited histone acetylation and administration of HDAC inhibitors, sodium butyrate or suberoylanilide hydroxamic acid (SAHA), protected against the αSyn-induced DA cell loss [197]. Valproic acid (VPA), another HDAC inhibitor selective for class I and IIa HDACs, has been clinically used for the treatment of bipolar mood disorder, schizophrenia, and convulsive seizures [198, 199]. Leng and Chuang [56] reported that VPA induced upregulation of αSyn through hyperacetylation of histone H3 in the *SNCA* promoter region in rat cerebellar granule cells and cortical neurons. The increased αSyn protein participated in neuroprotection against glutamate-induced excitotoxicity. By contrast, recent study indicated that VPA showed neuroprotective effect in rotenone-induced PD model rats [200]. In the minipump-mediated rotenone rats, monoubiquitinated αSyn increased its localization into the nuclei, suggesting that the monoubiquitinated αSyn functions in the nucleus to promote DA neuronal cell death. The intranuclear translocation of αSyn and subsequent DA cell death was attenuated by VPA treatment [200].

On the other hand, abnormally accumulating αSyn was found to induce ER stress via blocking the vesicular trafficking from ER to Golgi network [201]. In genome-wide screen, Rab guanosine triphosphatase YPT1 (a member of Rab subfamily that belongs to Ras superfamily) was identified to modify the cell toxicity of abnormal αSyn and associate with cytoplasmic αSyn inclusions in yeast cells. Transgenic expression of Rab1 (the murine YPT1 ortholog) rescued the loss of DA neuronal cells in *Drosophila* and *C. elegans* models of αSyn overexpression [201]. This research group indicated in the following experiments that αSyn disrupts localization of several Rab proteins [202]. Transduction of RAB8A, the human homolog of yeast Sec4p and a close paralog to Rab1, and another neuron-specific RAB3A that functions at the synapse were also able to provide substantial rescue against αSyn-induced DA neurodegeneration in *C. elegans* and primary midbrain culture [202].

Rapamycin is an allosteric inhibitor of mammalian target of rapamycin (mTOR), an intracellular serine/threonine protein kinase involved in various cellular processes including cell growth and proliferation, protein synthesis, and autophagy [reviewed in 203]. Rapamycin has been clinically used as immunosuppressant drug to prevent the graft rejection and is now extensively studied as promising anticancer agents because of its anti-proliferative properties [203]. Recent two reports indicated that systemic treatment with rapamycin protected DA neurons from death in MPTP mouse model of PD [204, 205]. The neuroprotective molecular cascade upregulated by systemic rapamycin was distinct in these two reports. One indicated that rapamycin blocked mTOR complex 1-induced upregulation of pro-cell death RTP801 protein, which inactivate mTOR complex 2-mediated phosphorylation of pro-survival Akt kinase [203, 204]. In the other report, mitochondria-derived ROS induced permeabilization of lysosomal membranes that resulted in accumulation of altered mitochondria and undegraded autophagosomes [203, 205]. The lysosomal membrane permeabilization also induced ectopic release of lysosomal proteases cathepsin B and D to the cytosol, which can cause the digestion of vital proteins or the activation of additional hydrolases, including caspases. All of these pathogenic events, including apoptotic cell death, can be attenuated by rapamycin treatment. In particular, rapamycin was shown to restore impairment of lysosome-mediated clearance of autophagosome, by boosting lysosomal biogenesis and promoting autophagolysosome formation [203, 205]. αSyn-transgenic mouse model of DLB and PD [136], which displayed elevation of mTOR, reduction of autophagy-related protein 7 (Atg7) levels, and the presence of abundant and abnormal autophagosomes, was also healed with rapamycin [206]. Intracerebral infusion of rapamycin into the lateral ventricle of αSyn-transgenic mice enhanced clearance of αSyn protein accumulating in neuronal cell bodies and synapses and redistribution to the axons, through upregulation of autophagy pathway. This study further indicated lentiviral vector-mediated Atg7 expression resulted in reduced accumulation of αSyn and amelioration of associated neurodegenerative alterations [206].

Neurotrophic factors including glial cell line-derived neurotrophic factor (GDNF) and its closely related family protein Neurturin have provided promising therapeutic effects in various animal neurotoxin models and phase I clinical trials for PD [reviewed in 207]. However, surprisingly, AAV or lentiviral vector-mediated GDNF delivery did not prevent DA neuronal cell loss induced by the virally overexpressed αSyn of wild-type or A30P mutant [208, 209]. The difference in neuroprotective efficacy of GDNF raises important issues pertinent to the relevance for the therapeutic use of GDNF and Neurturin in the patients with PD.

## Conclusion

αSyn has a central role in the pathogenesis of PD and other α-synucleinopathies, and a proper regulation of production, distribution, modification, and degradation of αSyn is crucial for neuronal functions and viability. Correction of the impairments in these multiple aspects of αSyn protein in its life cycle should provide disease modification remedies for the patients suffering from the devastating neurological disorders.

## Abbreviations

AAV: Adeno-associated virus
ACD: Autophagic cell death
αSyn: α-Synuclein
CaMKIIα: Calcium/calmodulin-dependent protein kinase IIα
CMA: Chaperone-mediated autophagy
CSPα: Cysteine-string protein-α
DA: Dopaminergic
DLB: Dementia with Lewy bodies
DMT1: Divalent metal transporter 1
Dnmt1: DNA methyltransferase 1
ER: Endoplasmic reticulum
GCase: Glucocerebrosidase
GDNF: Glial cell line-derived neurotrophic factor
HDAC: Histone deacetylase
Hsc70: Heat shock cognate 70
LRRK2: Leucine-rich repeat kinase 2
MPTP: 1-Methyl-4-phenyl-1,2,3,6-tetrahydropyridine
mTOR: Mammalian target of rapamycin
OB: Olfactory bulb
PCD: Programmed cell death
PD: Parkinson’s disease
PP2A: Phosphoprotein phosphatase A2
RIP: Receptor-interacting protein
RNS: Reactive nitrogen species
ROS: Reactive oxygen species
SN: Substantia nigra
SNAP-25: Synaptosomal-associated protein of 25 K
SNARE: Soluble *N*-ethylmaleimide-sensitive factor attachment protein receptor
SNpc: Substantia nigra pars compacta
TH: Tyrosine hydroxylase
UCH-L1: Ubiquitin carboxy-terminal hydrolase-L1
VPA: Valproic acid
